# Interactive removal of bacterial and viral particles during transport through low-cost filtering materials

**DOI:** 10.3389/fmicb.2022.970338

**Published:** 2022-08-04

**Authors:** Xijuan Chen, Liqiong Yang, Junjie Guo, Shuang Xu, Junzhen Di, Jie Zhuang

**Affiliations:** ^1^Key Laboratory of Pollution Ecology and Environmental Engineering, Institute of Applied Ecology, Chinese Academy of Sciences, Shenyang, China; ^2^School of Civil Engineering, Liaoning Technical University, Fuxin, China; ^3^College of Land and Environment, Shenyang Agricultural University, Shenyang, China; ^4^Department of Biosystems Engineering and Soil Science, Center for Environmental Biotechnology, Institute for a Secure and Sustainable Environment, The University of Tennessee, Knoxville, TN, United States

**Keywords:** co-transport, attachment, surface sites competition, iron filings, calcined magnesite

## Abstract

Pathogen filtration is critically important for water sanitation. However, it is a big challenge to balance removal efficiency and filtering material cost. In this study, we quantified the removal processes of a bacterial strain *Escherichia coli* 652T7 and a model bacteriophage MS2 (ATCC 15597-B1) during their transport through columns containing iron filings (IF), calcined magnesite (CM), natural ore limestone (OL) or corn stalk biochar (BC) under saturated flow conditions. Experimental results showed that 99.98, 79.55, 63.79, and 62.59% of injected *E. coli* 652T7 and 98.78, 92.26, 68.79, and 69.82% of injected MS2 were removed by IF, CM, OL, and BC, respectively. The differences in removal percentage were attributed to the disparities of the microorganisms and filtering materials in surface function groups, surface charges, and surface morphology. Transport modeling with advection-dispersion equation (ADE) and interaction energy calculation with extended Derjaguin, Landau, Verwey, and Overbeek (XDLVO) model indicated that *E. coli* 652T7 and MS2 were mostly removed *via* irreversible attachment. In IF columns, *E. coli* 652T7 promoted the transport of MS2 but not vice versa. In CM columns, MS2 facilitated the transport of *E. coli* 652T7 and vice versa at a less extent. Such changes were a combined result of attachment site competition, steric effect, and mechanical straining. We found that the sum of the removal percentages of the two microorganisms in their respective transport experiments were similar to those calculated from their co-transport experiments. This result suggests that the removals were mainly limited by the attachment sites in the filtering materials.

## Introduction

Waterborne diseases caused by emerging microbial contaminants are intensively reported worldwide (Verbyla and Mihelcic, 2015; Ran et al., 2019; Alegbeleye and Sant’Ana, 2020). Microbial contaminants include enteric viruses and pathogenic bacteria from various fecal sources. They can infiltrate into the subsurface through various pathways, such as improper discharge of wastewater, application of poultry manure, sludge landfill, and broken sewer lines (Sasidharan et al., 2017; Alegbeleye and Sant’Ana, 2020). Most of the microbial contaminants can travel a long distance and are relatively resistant and infectious at low doses (Fujita and Kobayashi, 2016; Mantha et al., 2017). The majority of waterborne diseases reported in the United States, the United Kingdom, Canada, and China were associated with groundwater and surface water contamination (Ligon and Bartram, 2016; Ambrosini et al., 2019). Therefore, understanding the removal and transport mechanisms of viral and bacterial pathogens is essential for the protection of public health.

A large number of experimental and theoretical studies have been carried out on microorganism transport through water and soil systems. The studies addressed the influences of air-water interface, solution chemistry (e.g., pH and ionic strength), soil properties (e.g., soil organic matter and pore structure), dissolved organic matter, and metal oxides. For example, Chrysikopoulos and Aravantinou (2014) studied the attachment of viruses onto quartz sand under batch experimental conditions, and the results demonstrated that temperature and grain size significantly affected virus attachment under static conditions. Xu et al. (2017) investigated the transport and retention behaviors of virus through metal oxide-removed and goethite-coated sand under saturated flow conditions, indicating that surface properties of porous media and viruses dominated the transport of viruses. Sasidharan et al. (2017) investigated the fate and transport of MS2, PRD1, and ΦX174 in a sandy limestone aquifer and found that pore-water velocity, residence time, and ionic strength were the key influencing factors. Yang et al. (2019) reported that advective chemotaxis, near-surface chemotaxis, and haptotaxis are coupled to determine the surface attachment of bacteria. Unfortunately, these studies focused on either bacteria or viruses and did not consider potential interactive removal and transport of bacteria and viruses when they co-exist, as occurring in natural environment.

Various materials such as zero-valent iron, limestone, biochar, and clay minerals have been reported to be able to adsorb and inactivate microorganisms (Kim et al., 2011; Chrysikopoulos and Syngouna, 2012; Sasidharan et al., 2017; Morshed et al., 2019). The attachment and inactivation are substantially controlled by inactive process between microorganisms and porous medium surfaces (Attinti et al., 2010; Shi et al., 2012; Syngouna and Chrysikopoulos, 2012; Park et al., 2014; Kokkinos et al., 2015; Zhang et al., 2021). However, these processes become much more complex in real situations of water treatment constructions and natural fields due to varying flow conditions and dynamic interactions between nano-sized viruses and micro-sized bacteria, which always co-exist. Therefore, it is essential to clarify how bacteria and viruses interact during their co-transport through filtration systems.

This study aimed to examine the removal and transport processes of bacteria and viruses in different low-cost filtering materials with an emphasis on microbial interactions during transport. The first set of column experiments focused on the separate removal and transport of bacteria only or viruses only, and the second set addressed simultaneous removal and transport when bacteria and viruses co-existed. Numerical simulations and interfacial interaction energy calculations were performed to quantify the mechanisms of removal and transport processes. The study provides valuable novel information on the removal potentials of different filtering materials and their influences on virus-bacterium interactions at solid-liquid interfaces under saturated flow conditions.

## Materials and methods

### Filtering materials

The filtering materials used in this study was iron filings (IF), calcined magnesite (CM), natural ore limestone (OL), and corn stalk biochar (BC). IF was obtained from a steel manufacture factory. CM and OL were collected from Liaoning Xiuyan Qinghua Mining Co., Ltd. (Yingkou, China). All the above three materials were sieved to 0.42–0.85 mm prior to use. Biochar was purchased from Shenyang Kalima Biochar Technology Development Co., Ltd. (Shenyang, China), and sieved through 2-mm mesh before use. The crystallographic structures and main compositions of the materials was analyzed by a computer-controlled X-ray diffractometer (Rigaku D/max 2400, Rigaku, Tokyo, Japan), and the data are provided in Supplementary Table 1. The surface morphology was analyzed by a scanning electron microscopy (SEM, Quanta 250, FEI, United States) coupled with energy dispersive X-ray Analyzer (EDX, AMETEK, United States) (Supplementary Figure 1). The EDX peaks indicated that the dominant component of IF, CM, OL, and BC was Fe (88.91%), MgO (89.1%), CaCO_3_ (90.46%), and carbon (95.82%), respectively.

The specific surface area of materials was determined using a Brunauer-Emmett-Teller (BET) surface area and porosity analyzer (ASAP 2460, Micromeritics, United States), the zeta potential was measured using laser particle analyzer (90Plus Zeta, Brookhaven, NY, United States), and water contact angles were measured with the sessile drop technique using a surface angle analyzer (Phoenix 300, Surface Electro Optics, Ansan, Korea) at 20°C. The characteristics of IF, CM, OL, and BC are presented in Table 1.

**TABLE 1:** Basic characteristics of iron filings (IF), calcined magnesite (CM), natural ore limestone (OL), corn stalk biochar (BC), and microorganisms.

Material/Microorganism	Size	Specific surface area	Zeta potential	Contact angle
		
		(m^2^ g^–1^)	(mV)	(°)
IF	0.45∼0.85 mm	0.14	–8.02 ± 0.61	84 ± 1.72
CM	0.45∼0.85 mm	18.7	4.40 ± 0.52	10.62 ± 1.53
OL	0.45∼0.85 mm	0.25	–21.09 ± 1.37	19.89 ± 1.31
BC	<2 mm	2.8	–42.5 ± 3.14	106.5 ± 1.5
*E. coli* 652T7	1.2 ± 0.2 μm	–	–43.26	50 ± 1.21
MS2	26 nm	–	–21.44	32 ± 0.87

### Microorganisms

The experiments were performed using motile bacterial strain *Escherichia coli* 652T7 and a model bacteriophage MS2 (ATCC 15597-B1). *E. coli* 652T7 is a gram-negative bacterium of a particle size of 0.4–0.7 × 1–3 μm with flagella on the surface, and was obtained from the University of Tennessee’s Center for Environmental Biotechnology, United States. MS2 is an icosahedral, positive-sense single-stranded RNA virus that infects specific bacteria and is harmless to human health. MS2 together with its host bacteria *E. coli* ATCC 15597 were obtained from the American Type Culture Collection (ATCC).

Both *E. coli* 652T7 and MS2 strains were individually cultured in 100 mL of Luria-Bertani broth (LB) supplemented with 50 mg L^–1^ kanamycin (CAS 133-92-6) in a rotary shaker (HZQ-X160) at 160 rpm and 37°C for 19.5 h. Cells were then cultured by centrifugation (2,000 g, 20 min, 4^°^C), and the suspension was filtered using a 0.45-μm membrane before storage at 4^°^C. All the solutions and utensils were sterilized for 30 min (121°C, 103 kPa) prior to further usage. Cell size and zeta potentials were measured in triplicate in column experimental background solution (containing 2 mM NaCl) at 25^°^C using a laser particle analyzer (90 Plus Zeta, Brookhaven, NY, United States). Contact angle of water drops on the bacterial lawns were measured by goniometer microscope (Krüss GmbH, Germany) and used to analyze cell-surface hydrophobicity (Table 1). *E. coli* 652T7 and MS2 were measured by quantifying colony-forming units (CFUs) and plaque forming unit (PFUs) (Adams, 1959), with detection limits of 10 CFUs or PFUs per plate. In this study, we unified these two units into N.

### Column experiments

The column experiments were performed to investigate the removal and transport of microorganisms in different materials. The column system was set up as shown in Supplementary Figure 1, which consisted of a stainless-steel column (10 cm in length, 1 cm in inner diameter), a piston pump (LC-16, Shimadzu Corporation, Kyoto, Japan), and a fraction collector (CF2, Spectrum Chemical Mfg. Corp., CA, United States). The upper and lower end of the column was fitted with a hydrophilic nylon membrane (30 μm in mesh size) in order to avoid loss of filtering materials during the transport experiments. The material was packed into the column at ∼ 1 cm increments with tapping. After packing, the column was flushed with high pressure CO_2_ gas for ∼ 3 h to displace trapped air and then with de-aerated and sterilized background solution (2 mM NaCl solution, pH 7.5) at a flow rate of 0.1 mL min^–1^ for 20 pore volumes in order to standardize the chemical conditions and establish a steady-state flow condition. Each experiment was started by introducing 30 pore volumes (∼ 15 h) of de-aerated influent solution, which contained 2 mM NaCl, 30 mg L^–1^ NaBr (conservative tracer), and microorganisms (respective transport experiment: 10^8^ N mL^–1^ *E. coli* 652T7 or 10^8^ N mL^–1^ MS2; co-transport experiment: 10^8^ N mL^–1^ *E. coli* 652T7 + 10^8^ N mL^–1^ MS2) for 30 pore volumes. The influent solution was stirred by a magnetic stirrer to ensure a uniform concentration of the microorganisms, while their concentrations were measured hourly during the column experiments. The average hydraulic retention time (HRT) of the influent solution in the material was set to 0.5 h with a pore water velocity of 21–22 cm h^–1^. The effluent samples were collected at regular time intervals using a fraction collector. The column experiments were performed in replicates to examine the reproducibility of results. Concentration of bromide was measured in replicates using Ion Chromatography (DIONEX, ICS-5000), and the concentrations of *E. coli* 652T7 and MS2 were determined in triplicates by plate counting after serial dilution.

### Transport modeling

The breakthrough of bromide under steady-state saturated flow conditions was simulated using the Hydrus-1D software with a one-dimensional ADE:


(1) $$\frac{\partial C}{\partial t}=D\,\frac{\partial^{2}C}{\partial z^{2}}-v\,\frac{\partial C}{\partial z}$$

where *C* is bromide concentration in liquid phase (mg L^–1^), *t* is time (h), *v* is pore water velocity (cm h^–1^), *D* is fitted dispersion coefficient (cm^2^h^–1^), and *z* is the distance from the inlet (cm).

The porous media used in this study are geochemically heterogeneous and composted of different surfaces with both favorable and unfavorable sites for the attachment of bacteria and viruses. Therefore, the attachment can be assumed as two-site kinetic attachment-detachment processes, and the ADE was modified as follows:


(2) $$\frac{\partial C}{\partial t}+\frac{\rho }{\theta }\,\frac{\partial S_{1}}{\partial t}+\frac{\rho }{\theta }\,\frac{\partial S_{2}}{\partial t}=D\,\frac{\partial^{2}C}{\partial z^{2}}-v\,\frac{\partial C}{\partial z}-\mu_{l}\,C$$

where C is the bacterium or virus concentration (N mL^–1^) in the effluent, ρ (g cm^–3^) is the bulk density of the porous filtering materials, θ (dimensionless) is the porosity, S_1_ (N g^–1^), and S_2_ (N g^–1^) are the solid phase concentrations of microorganisms associated with attachment sites 1 and 2, respectively, μ_l_ (h^–1^) denotes microorganism decay in the liquid phase, and D (cm^2^ h^–1^) was obtained by fitting the bromide data to Equation (1).

Subgroups to Equation (2) are Equations (3) and (4) are:


(3) $$\frac{\rho }{\theta }\,\frac{\partial S_{1}}{\partial t}=k_{\mathrm{att1}}\,{\text{C-k}}_{\det1}\,S_{1}\,\frac{\rho }{\theta }-\mu_{\text{s}}\,\rho \,S_{1}$$

(4) $$\frac{\rho }{\theta }\,\frac{\partial S_{2}}{\partial t}=k_{\mathrm{att2}}\,{\text{C-k}}_{\det2}\,S_{2}\,\frac{\rho }{\theta }-\mu_{\text{s}}\,\rho \,S_{2}$$

where k_att_ (h^–1^) is the first-order attachment coefficient, k_det_ (h^–1^) is the first-order detachment coefficient, subscripts 1 and 2 refer to the attachment sites, and μ_*s*_ (h^–1^) represent inactivation/degradation processes in the solid phases.

### Extended Derjaguin, Landau, Verwey, and Overbeek and steric interactions

The XDLVO theory was used to estimate the total surface potential energy (Φ_XDLVO_) based on the summation of potential energies contributed by van der Waals (Φ_Vdw_), electrostatic double layer (Φ_Edl_), Lewis acid-base (Φ_AB_), and Born interactions (Φ_Born_). The input parameters are shown in Table 2. The Equations are given below:


(5) Φ⁢X⁢D⁢L⁢V⁢O=Φ⁢V⁢d⁢w+Φ⁢E⁢d⁢l+Φ⁢B⁢o⁢r⁢n+Φ⁢A⁢B

**TABLE 2:** Experimental and fitted model parameters of respective transport (RT) and co-transport (CT) column experiments.

Microorganism	Material	D (cm^2^h^–1^)	K_*att1*_ (h^–1^)	K_*det1*_ (h^–1^)	K_*att2*_ (h^–1^)	K_*det2*_ (h^–1^)	Removal (%)	*R* ^2^
*E. coli* 652T7-RT	IF	1.13	30.91	0.01	34.28	0.01	99.98 ± 3.50	0.88
	CM	1.34	9.20	0.25	11.67	0.14	81.69 ± 2.55	0.88
	OL	0.52	5.75	0.09	7.47	0.07	63.79 ± 2.55	0.89
	BC	0.51	0.98	0.00	2.43	0.03	62.59 ± 1.88	0.93
*E. coli* 652T7-CT	IF	1.27	23.12	0.03	26.49	0.02	99.86 ± 3.50	0.98
	CM	2.71	3.01	0.69	7.29	0.29	43.62 ± 1.40	0.97
MS2-RT	IF	2.11	23.15	0.14	25.67	0.13	98.78 ± 3.46	0.95
	CM	1.08	10.83	0.19	12.94	0.11	92.26 ± 2.95	0.90
	OL	0.65	5.72	0.08	7.45	0.06	68.79 ± 2.75	0.95
	BC	0.33	1.30	0.02	2.93	0.03	69.82 ± 2.09	0.95
MS2-CT	IF	1.69	13.47	0.15	15.21	0.12	93.49 ± 3.27	0.94
	CM	1.68	11.28	0.08	13.15	0.06	95.68 ± 3.06	0.93

D, dispersion coefficient (cm^2^h^–1^); k_att1_, first-order attachment coefficient on site 1; k_att2_, first-order attachment coefficient on site 2; k_det1_, first-order detachment coefficient on site 1; k_det2_, first-order detachment coefficient on site 2; IF, iron filings; CM, calcined magnesite; OL, natural ore limestone; BC, corn stalk biochar.

The potential energies of van der Waals (Φ_Vdw_), electrostatic double layer (Φ_Edl_), Lewis acid-base (Φ_AB_) and Born interactions (Φ_Born_) can be calculated using the following Equations:


(6) $$\Phi_{\text{Vdw}}=-\frac{A_{123}\,r}{6\,h}\,\left[1+\left(\frac{14\,h}{\lambda }\right)\right]-1$$

(7) $$\Phi_{\text{Edl}}=\pi r\varepsilon_{0}\varepsilon_{\text{r}}\left\{2\varphi_{1}\varphi_{2}\ln\left[\frac{1+\exp\left(-k\,h\right)}{1-\exp\left(-k\,h\right)}\right]+(\varphi_{1}^{2}+\varphi_{2}^{2}\right)\ln\left[1-\exp\left(-2kh\right)\right]\}$$

(8) $$k=\sqrt{\frac{2\,N_{\text{A}}\,e^{2}\,l}{\varepsilon_{0}\,\varepsilon_{\text{r}}\,k_{\text{B}}\,T}}$$

(9) $$\Phi_{\text{Born}}=\frac{A_{123}\,\sigma_{\text{Born}}^{6}}{7560}\,\left[\frac{8\,r_{\text{p}}+h}{{\left(2\,r_{p}+h\right)}^{7}}+\frac{6\,r_{\text{p}}-h}{h^{7}}\right]$$

(10) $$\Phi_{\text{AB}}=2\,\pi \,r_{\text{p}}\,\lambda_{\text{AB}}\,\Phi_{\mathrm{AB}\left(h=h_{0}\right)}\,\exp\left[\frac{h_{0}-h}{\lambda_{\text{AB}}}\right]$$

where *r* (m) is the radius of microorganisms, *h* (m) is the separation distance between microorganism and filtering material surfaces, λ (m) is the characteristic wavelength of the dielectric, λ_AB_ (nm) is the decay length of water (taken as 1 nm), ε_0_ (C^2^t^2^ ML^–2^) is the permittivity of free space, ε_r_ is the relative dielectric constant of the suspending liquid, *k*(m^–1^) is the inverse of the diffuse layer thickness, I is the ionic strength (mm), N_A_ (6.02 × 10^23^ L mol^–1^) is the Avogadro number, e (–1.60 × 10^–19^ C) is the elementary charge, K_B_ (1.38 × 10^–23^ J K^–1^) is Boltzmann constant, T (293 K) is absolute temperature, A_123_ is the Hamaker constant, which represents microorganisms interactions with various interfaces. Parameters φ_1_ and φ_2_ are the surface potentials of microorganisms and particles, respectively, and σ_Born_ (m) represents the Born collision parameter.

The *Φ_*AB(h* = *h0*)_* (J m^–2^) is the Lewis acid-base free interaction energy between two surfaces at *h* = *h*_0_, which can be calculated by the following Equations:


(11) $$\Phi_{AB\left(h=h_{0}\right)}=-\frac{K_{123}}{2\,\pi \,h_{0}\,\lambda_{A\,B}}$$

(12) $$\log K_{123}=-7.0\,\left(\frac{\cos\beta_{1}+\cos\beta_{3}}{2}\right)-18.0$$

where K_123_ (J) is the hydrophobic force constant, and β_1_ (°) and β_3_ (°) are the water contact angles of microorganisms and filtering materials, respectively.

## Results and discussion

### Respective removal and transport of virus and bacterium

**FIGURE 1:**
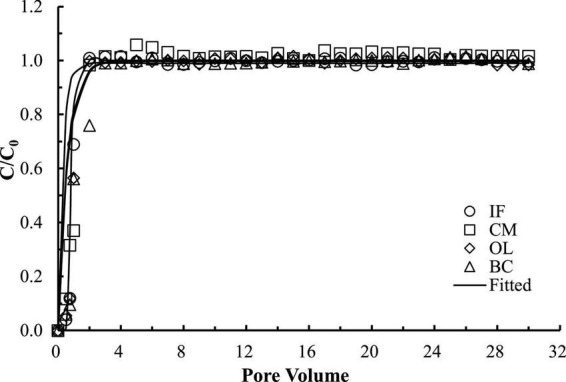
Transport of bromide ion through different porous materials. IF, iron filings; CM, calcined magnesite; OL, natural ore limestone; BC, corn stalk biochar. The lines are the modeling results with advection-dispersion equation using the Hydrus-1D software.

**FIGURE 2:**
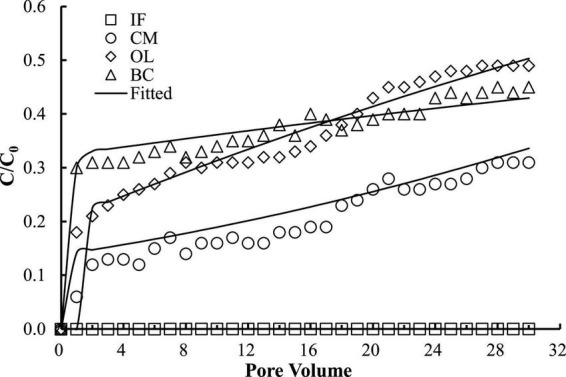
Respective transport of *E. col*i 652T7 in different materials. IF, iron filings; CM, calcined magnesite; OL, natural ore limestone; BC, corn stalk biochar. The lines are the modeling results with advection-dispersion equation using the Hydrus-1D software.

**FIGURE 3:**
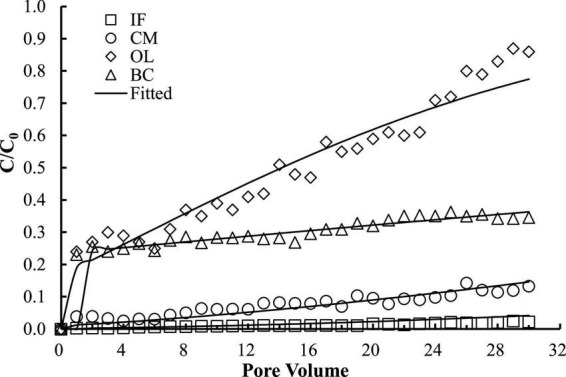
Respective transport of MS2 in different materials. IF, iron filings; CM, calcined magnesite; OL, natural ore limestone; BC, corn stalk biochar. The lines are the modeling results with advection-dispersion equation using the Hydrus-1D software.

The good reproducibility of the bromide breakthrough curves indicated that the hydrological conditions remained stable and consistent during all of the column experiments (Figure 1). The breakthrough curves of microorganisms from the columns packed with different filtering materials are demonstrated in Figures 2, 3. The microorganisms broke through the column at ∼1 pore volume, and their initial and maximal removals varied with filtering materials. The fitted parameters in Table 2 indicated that IF was most effective in removing *E. coli* 652T7 (99.98%), followed by CM (81.69%), OL (63.79%), and BC (62.59%). A similar trend was observed for MS2, with removal percentages of 98.78% by IF, 92.26% by CM, 68.79% by OL, and 69.82% by BC. These results suggest that microorganism removal was favorable in porous materials of the order of iron > magnesium > calcium > carbon materials.

The one-dimensional advection-dispersion model provided a good description for both breakthrough and retention profiles of microorganisms according to the coefficients of determination, *R*^2^, which ranged between 0.88 and 0.98 (Table 2). The estimated values of the first-order attachment coefficients (*K*_*att1*_ and *K*_*att2*_) were highest in IF columns (23.15–34.28 h^–1^), intermediate in CM columns (9.2–12.94 h^–1^), and lower in OL and BC columns (0.98–7.47 h^–1^). Meanwhile, the low values of the first-order detachment coefficients (*K*_*det1*_ and *K*_*det2*_) suggested a long residence time of the microorganisms on the material surfaces (Schijven and Šimùnek, 2002; Katzourakis and Chrysikopoulos, 2014).

**FIGURE 4:**
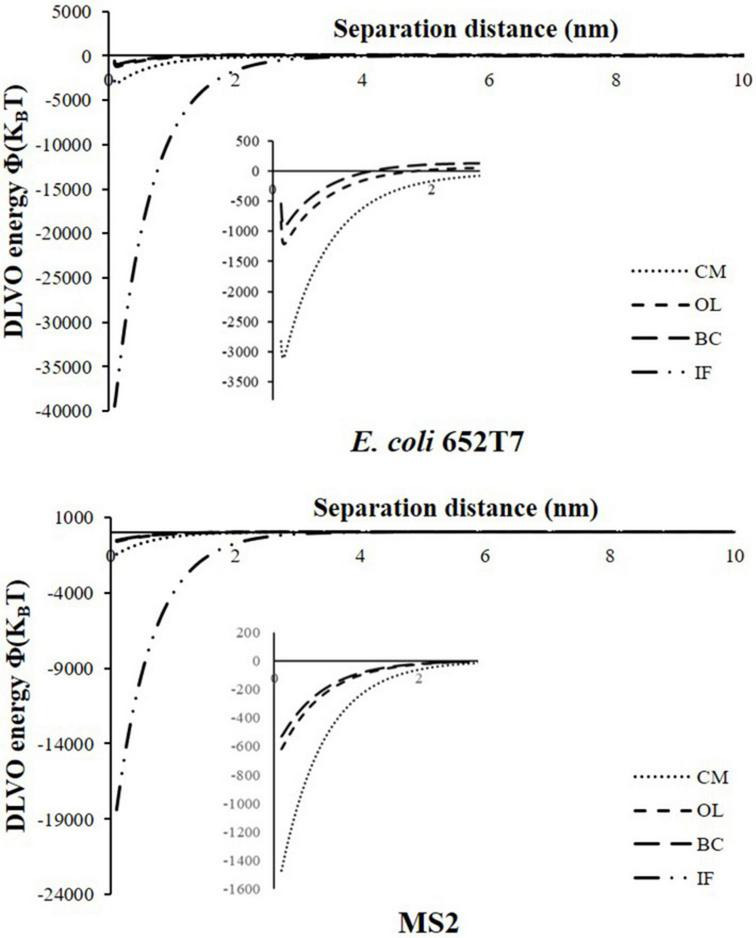
Predicted potential energy profiles of Φ_XDLVO_ for microbial particles material surfaces as a function of the separation distance. IF, iron filings; CM, calcined magnesite; OL, natural ore limestone; BC, corn stalk biochar.

The removal behaviors of microorganisms were further evaluated according to the interaction energy profiles estimated using the XDLVO model. Figure 4 shows the calculated total energies (*Φ_XDLVO_*) for interactions between microorganisms and different materials. Table 3 provides the calculated values of primary minima (*Φ_*min1*_*), secondary minima (*Φ_*min2*_*), and primary energy barriers (*Φ_*max1*_*) of the XDLVO energy. As expected, the calculated XDLVO interaction forces were attractive in all cases. The low value of *Φ_*min1*_* indicated that the attachment of microorganisms on the material surfaces were irreversible, whereas the non-existence of *Φ_*max1*_* and *Φ_*min2*_* suggested that attractive forces dominated the attachment of microorganisms (Table 3). The magnitudes of *Φ_*min1*_* were greater in IF column than CM, OL, and BC columns. The *Φ_*max1*_* were observed in the energy profiles of microorganism interactions with OL and BC, and the results indicated that sufficient kinetic energy is needed to overcome the potential energy barrier before attachment onto OL and BC surfaces. Worth noting is that *Φ_*max1*_* value is slightly higher on OL than BC surfaces. This difference explains the relatively lower attachment of bacteria on OL and BC. The results of the XDLVO interaction energies revealed that *E. coli* 652T7 and MS2 were more prone to attach on the IF and CM than on other materials with the order of IF > CM > OL > BC. It should be noted that both transport experiments and XDLVO calculations predicted the same trend of the removals of the two microorganisms.

**TABLE 3:** Estimated values of XDLVO interaction energy parameters.

Material-microorganism	*Φ_*AB(h* = *h0)*_* (J m^–2^)	Primary minima (K_*B*_T)	Secondary minima (K_*B*_T)	Primary energy barrier (K_*B*_T)
IF- *E. coli* 652T7	–0.64	–39465.54	NA	NA
CM- *E. coli* 652T7	–0.05	–3098.28	NA	NA
OL- *E. coli* 652T7	–0.02	–1207.19	NA	61.60
BC- *E. coli* 652T7	–0.02	–924.81	NA	131.46
IF-MS2	–17.64	–18381.40	NA	NA
CM-MS2	–1.41	–1468.63	NA	NA
OL-MS2	–0.60	–615.99	NA	0.47
BC-MS2	–0.51	–527.16	NA	0.99

Φ_AB(h = h0)_, Lewis acid-base free interaction energy (J m^–2^); IF, iron filings; CM, calcined magnesite; OL, natural ore limestone; BC, corn stalk biochar.

The removal of microorganisms by filtering materials was attributed to the retention and inactivation caused by physical and chemical interactions (Bellou et al., 2015; Zhang and Zhang, 2015; Jiang et al., 2018). *E. coli* 652T7 and MS2 showed the highest removal percentage of 98.78–99.98% in IF due likely to the inactivation caused by IF oxidation process (Lee et al., 2008). Kim et al. (2010, 2011) reported that nano zero-valent iron (nZVI) rapidly inactivated *E. coli* and MS2 in both presence and absence of oxygen due to the generation of intracellular oxidants, which damaged the membrane integrity and respiratory activity of microorganisms. The antibacterial effect of magnesium oxide is mainly driven by the strong adsorption forces when microorganisms contact the oxide surfaces. The magnesium oxide surfaces (especially the spikes) can deform and rupture the cell wall and membrane, leading to leakage of intracellular substances and eventually death of bacteria (Leung et al., 2014). Jeevanandam and Klabunde (2002) reported that there are active adsorption sites on the surfaces of magnesium oxide, such as Lewis acid Mg^2+^ sites, Lewis base O^2–^ sites, lattice-bound hydroxyl groups, free hydroxyl groups, and anion and cation holes, all of which can provide adsorption sites for functional groups of viral surface proteins.

Surface charges significantly influence the attachment of microorganisms to materials. In this study, IF and CM had relatively higher zeta potential than OL and BC (Table 2). As a result, the electric attractions between the microorganisms and the oxide surfaces enhanced, causing high removal of *E. coli* 652T7 in IF (99.98%) and in CM (79.55%) and likewise high removal of MS2 in IF (98.78%) and in CM (92.26%). In comparison, OL and BC exhibited much lower removal percentages for both *E. coli* 652T7 and MS2 (Figures 2, 3), with removal percentages of 63.79–68.79% in OL and 62.59–69.82% in BC. This removal differences are related to the lower zeta potentials, which were –21.086 ± 1.37 mV for OL and –42.5 ± 3.14 mV for BC. The low zeta potentials enhanced electrostatic repulsion and was unfavorable to the interactions of the microorganisms with OL and BC.

Surface morphologies can influence the attachment and transport of colloids by hindering water film spreading and altering the water cohesion and capillarity (Cunha and Gandini, 2010; Zhuang et al., 2010). According to the measured water contact angles (Table 1), three materials (IF, CM, and OL) are characterized as wetting surfaces (water contact angle < 90°C), suggesting that they are conducive to attachment compared with non-wetting BC surface (water contact angle > 90°C) (Chrysikopoulos and Syngouna, 2012; Yuan et al., 2017). The more negative values of *Φ_*AB(h* = *h0)*_* of *E. coli* 652T7 (–0.64 J/m^2^) and MS2(–17.64 J/m^2^) on the IF surfaces suggest that the attachment of hydrophilic microorganisms onto IF was greater than that onto CM, OL and BC (Table 3). In comparison, the calculated *Φ_*AB(h* = *h0)*_* values of *E. coli* 652T7 on CM (–0.05 J/m^2^), MS2 on CM (–1.41 J/m^2^), *E. coli* 652T7 on OL (–0.05 J/m^2^), and MS2 on CM (–0.60 J/m^2^) were small, indicating that the hydrophobic interactions of microorganisms with CM and OL were not significant because CM and OL have smaller water contact angles. Chrysikopoulos and Syngouna (2012) indicated that hydrophobic interactions between two approaching surfaces become influential when the water contact angles are greater than 65°. Syngouna and Chrysikopoulos (2013) reported a similar finding that *Φ_*AB(h* = *h0)*_* was not significant for MS2 and ΦX174 adhesion to the surfaces of glass beads.

Surface properties and particle sizes of microorganisms also determine their removals in porous media. *E. coli* 652T7 has a zeta potential of –45.1 ± 2.8 mV, which was lower than MS2 (–21.44 ± 1.14 mV). Thus, *E. coli* 652T7 exhibited higher electrostatic repulsion from negatively charged materials such as OL and BC, leading to its slightly lower removal percentage (62–64%) compared to MS2 (67–70%). This is consistent with previous result that the removal was positively correlated to surface charges and roughness (Park and Kim, 2015; Morshed et al., 2019). Another mechanism filtering microorganisms in porous media is mechanical straining, which refers to the retention of microbial particles in the smallest pore space formed between material grains or narrow channels (Porubcan and Xu, 2011; Bradford et al., 2013; Mushenheim et al., 2016). Bradford and Bettahar (2006) and Bradford et al. (2007) established time-dependent straining model. They suggested that straining force increased with the ratio of microorganism diameter (d_c_) to material grain diameter (d_50_) with a threshold value of 0.003. In this study, the diameter ratio of *E. coli* 652T7 to material grain (d_c_/d_50_) was 0.014–0.027 for IF, CM, and OL and 0.0006 for BC. The d_c_/d_50_ values of MS2 in IF, CM, and OL ranged between 3.2 × 10^–7^ and 5.8 × 10^–7^ for IF, CM, and OL, and 0.13 × 10^–6^ for BC. Therefore, mechanical straining played a key role in the removal of *E. coli* 652T7 in IF, CM, and OL, but was not a significant mechanism for MS2 removal.

### Interactive removal and transport of co-existing virus and bacterium

**FIGURE 5:**
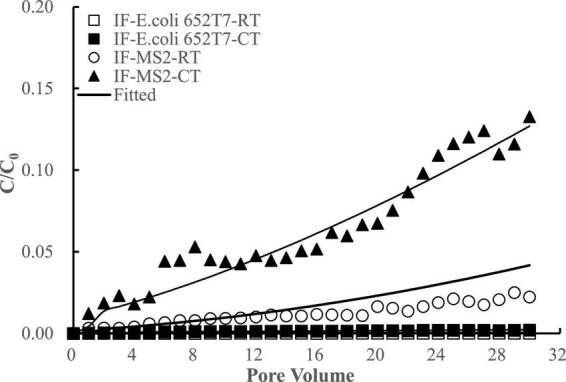
Respective transport (RT) and co-transport (CT) of *E. coli* 652T7 in iron filings (IF). The lines are the modeling results with advection-dispersion equation using the Hydrus-1D software.

Considering potential competition of viral and bacterial particles for the attachment sites on the filtering materials, we conducted experiments to investigate the co-transport and interactive removal behaviors of *E. coli* 652T7 and MS2 in IF and CM. The results showed that *E. coli* 652T7 promoted the transport of MS2 through IF (Figure 5). The breakthrough percentage of MS2 increased from ∼2 to ∼7% when *E. coli* 652T7 co-existed at a concentration of 2.25 × 10^8^ N mL^–1^. However, MS2 did not significantly influence the transport of *E. coli* 652T7. This result is attributed to the differences in zeta potential and particle size between *E. coli* 652T7 and MS2. Compared to MS2, *E. coli* 652T7 exhibited a stronger competition for the electropositive attachment sites as indicated by the larger *K*_*att1*_ and *K*_*att2*_ of *E. coli* 652T7 than MS2. As a result, once *E. coli* 652T7 was attached onto the IF surface to occupy a relatively larger surface area, it blocked MS2 attachment *via* steric hindrance effect, thereby facilitating the breakthrough of MS2 (Afrooz et al., 2008; Xu et al., 2017). It is interesting to note that the sum of the removal of *E. coli* 652T7 and MS2 calculated from the respective transport experiments, which was 1,765 ± 62 N μg^–1^ (*E. coli* 652T7: 863 ± 30 N μg^–1^; MS2: 902 ± 32 N μg^–1^), was similar to that calculated from the co-transport experiment, which was 1,863 ± 65 N μg^–1^ (*E. coli* 652T7: 1,078 ± 38 N μg^–1^; MS2: 785 ± 27 N μg^–1^) (Table 4). This result suggested that the IF provided same total surface area for microbial attachment under both the respective and co-transport experiments.

**TABLE 4:** Removal of microorganisms in respective transport (RT) and co-transport (CT) experiments.

Microorganism	Material	Input concentration (N mL^–1^)	Removal (%)	Removal (N μg^–1^)
*E. coli* 652T7-RT	IF	1.80E+08	99.98 ± 3.50	863 ± 30
	CM	1.79E+08	81.69 ± 2.55	1,613 ± 52
*E. coli* 652T7-CT	IF	2.25E+08	99.86 ± 3.50	1,078 ± 38
	CM	1.99E+08	43.62 ± 1.40	983 ± 31
MS2-RT	IF	1.90E+08	98.78 ± 3.46	902 ± 32
	CM	1.29E+08	92.26 ± 2.95	1,352 ± 43
MS2-CT	IF	1.75E+08	93.49 ± 3.27	785 ± 27
	CM	1.79E+08	95.68 ± 3.06	1,944 ± 62

IF, iron filings; CM, calcined magnesite; OL, natural ore limestone; BC, corn stalk biochar.

**FIGURE 6:**
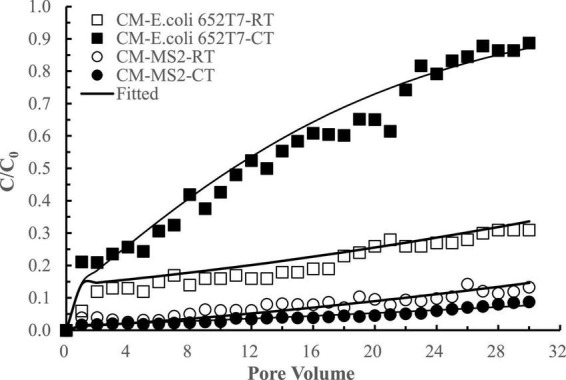
Respective transport (RT) and co-transport (CT) of *E. coli* 652T7 and MS2 in calcined magnesite (CM). The lines are the modeling results with advection-dispersion equation using the Hydrus-1D software.

The interactions between *E. coli* 652T7 and MS2 in the CM columns were different from those in the IF columns (Figure 6). When *E. coli* 652T7 and MS2 co-existed, the removal of *E. coli* 652T7 decreased from ∼80 to ∼44% while the removal of MS2 increased from ∼92 to ∼96%. Such interactions between *E. coli* 652T7 and MS2 is attributed to two processes. One is the competition for limited attachment sites. The fitted parameters in Table 2 showed that MS2 had larger attachment coefficients (*K*_*att1*_ and *K*_*att2*_) and higher affinity than *E. coli* 652T7, leading to more efficient competition of MS2 over *E. coli* 652T7 on the CM surfaces. The other is the mechanical straining strengthened by the high surface roughness of CM (Supplementary Figure 2). The straining effect was stronger for the micro-sized *E. coli* 652T7 than nano-sized MS2, leading to decrease in surface attachment of *E coli* 652T7 than MS2 (Katzourakis and Chrysikopoulos, 2014). In addition, the sum of removal of the two microorganisms calculated from their respective transport experiments (total 2,965 ± 95 N μg^–1^ with *E. coli* 652T7: 1,613 ± 52 N μg^–1^ and MS2: 1,352 ± 43 N μg^–1^) was similar to that calculated from their co-transport experiment (total 2,927 ± 93 N μg^–1^ with *E. coli:* 652T7: 983 ± 31 N μg^–1^ and MS2: 1,944 ± 62 N μg^–1^) (Table 4). This result suggested the removal of *E. coli* 652T7 and MS2 were dominantly determined by the limited attachment sites of CM.

The difference in the interactive removal of the two microorganisms in IF and CM materials is apparently related to their surface morphology (Table 1 and Supplementary Figure 2). IF has relatively flat surface with low roughness (i.e., less porosity or larger pores) and is thus favorable to bind micro-sized *E. coli* 652T7 than nano-sized MS2. As a result, *E. coli* 652T7 facilitated MS2 transport *via* steric effect. In comparison, CM has a very high surface roughness with fine spikes (i.e., high porosity and smaller pores). The spikes and smaller pores favored the access and attachment of nano-sized MS2 than micro-sized *E. coli* 652T7. As a result, the viruses were preferentially attached to modify the spikes, reducing bacterial attachment.

## Conclusion

The respective and co-transport of a bacterium (*E. coli* 652T7) and a virus (MS2) through different waste porous materials was investigated through flow-through column experiments and evaluated using the ADE and XDLVO models. Characteristics of porous materials are the key factors influencing the removal and transport of the two microorganisms. Both *E. coli* 652T7 and MS2 were favorably removed in the materials with the order of IF > CM > OL > BC. The greater removal by IF and CM than by OL and BC was attributed to surface charges and surface roughness. ADE simulation on the respective transport process of the two microorganisms suggested that attachment affinities of *E. coli* 652T7 and MS2 to materials were in the order of IF > CM > OL > BC, while XDLVO computation indicated that the two microorganisms were removed mostly *via* irreversible attachment mechanisms. In the co-transport scenario, *E. coli* 652T7 and MS2 competed for limited attachment sites. Microorganisms with higher affinity to materials exhibited stronger competition for the attachment sites, leading to their increased removal and decreased transport and meanwhile facilitating the transport of other microorganisms with lower affinity. In addition, the surface characteristics of materials, such as surface charges and morphology, could be modified after microorganism’s attachment to hinder the attachment of other microorganisms with weak competition ability. Our results demonstrated that viral removal was mainly controlled by electrostatic interactions at solid-liquid interfaces while bacterial removal was dominated by both electrostatic forces and mechanical straining. The filtering materials provided limited attachment sites in retaining microorganisms, and thus the removal of microorganisms was dominated by surface properties and total surface area of the filtering materials.

## Data availability statement

The original contributions presented in this study are included in the article/Supplementary material, further inquiries can be directed to the corresponding author/s.

## Author contributions

XC and JZ: conceptualization. XC, LY, and SX: methodology. XC and JG: data gathering and funding acquisition. LY and SX: data analysis. JZ and JD: validation and project administration. XC: writing—original draft preparation. XC, JD, and JZ: writing—review and editing. All authors contributed to the article and approved the submitted version.
