# Supplementary material for: Interactive removal of bacterial and viral particles during transport through low-cost filtering materials
Source: Front Microbiol. 2022 Aug 4;13:970338. doi: 10.3389/fmicb.2022.970338 (PMC9386502; doi:10.3389/fmicb.2022.970338)
Supplement: Supplementary file 1 [file Data_Sheet_1.docx]

**Table S1**. Composition of waste porous materials analyzed by X-ray diffraction.

| **Iron filings (IF)** | | | | | | | | | | | | | | | | | | | |
| --- | --- | --- | --- | --- | --- | --- | --- | --- | --- | --- | --- | --- | --- | --- | --- | --- | --- | --- | --- |
| Composition | Fe | SiO_2_ | | Fe_2_O_3_ | | MgO | | SO_3_ | | Al_2_O_3_ | | P_2_O_5_ | | MnO | | Tb_4_O_7_ | | Co_3_O_4_ | Gd_2_O_3_ |
| Mass (%) | 88.19 | 4.47 | | 2.828 | | 1.55 | | 1.54 | | 0.71 | | 0.7 | | 0.58 | | 0.46 | | 0.26 | 0.25 |
| **Calcined magnesite (CM)** | | | | | | | | | | | | | | | | | | | |
| Composition | MgO | Na_2_O | | SO_3_ | | SiO_2_ | | P_2_O_5_ | | CaO | | Fe_2_O_3_ | | MnO | | K_2_O | | La_2_O_3_ | CeO_2_ |
| Mass (%) | 89.1 | 3.61 | | 2.54 | | 1.4 | | 1.27 | | 1.26 | | 0.68 | | 0.84 | | 0.02 | | 0.02 | 0.02 |
| **Natural ore limestone (OL)** | | | | | | | | | | | | | | | | | | | |
| Composition | CaCO_3_ | SiO_2_ | | CaO | | MgO | | SO_3_ | | Al_2_O_3_ | | K_2_O | | Fe_2_O_3_ | | P_2_O_5_ | | Dy_2_O_3_ | SrO |
| Mass (%) | 90.46 | 3.6 | | 3.26 | | 2.38 | | 1.38 | | 0.68 | | 0.46 | | 0.45 | | 0.38 | | 0.07 | 0.05 |
| **Corn stalk biochar (BC)** | | | | | | | | | | | | | | | | | | | |
| Composition | C | | K_2_O | | SO_3_ | | Fe_2_O_3_ | | Al_2_O_3_ | | CaO | | TiO_2_ | | Na_2_O | | P_2_O_5_ | | |
| Mass (%) | 95.82 | | 2.62 | | 0.45 | | 0.63 | | 0.58 | | 0.98 | | 0.05 | | 0.05 | | 0.05 | | |

**
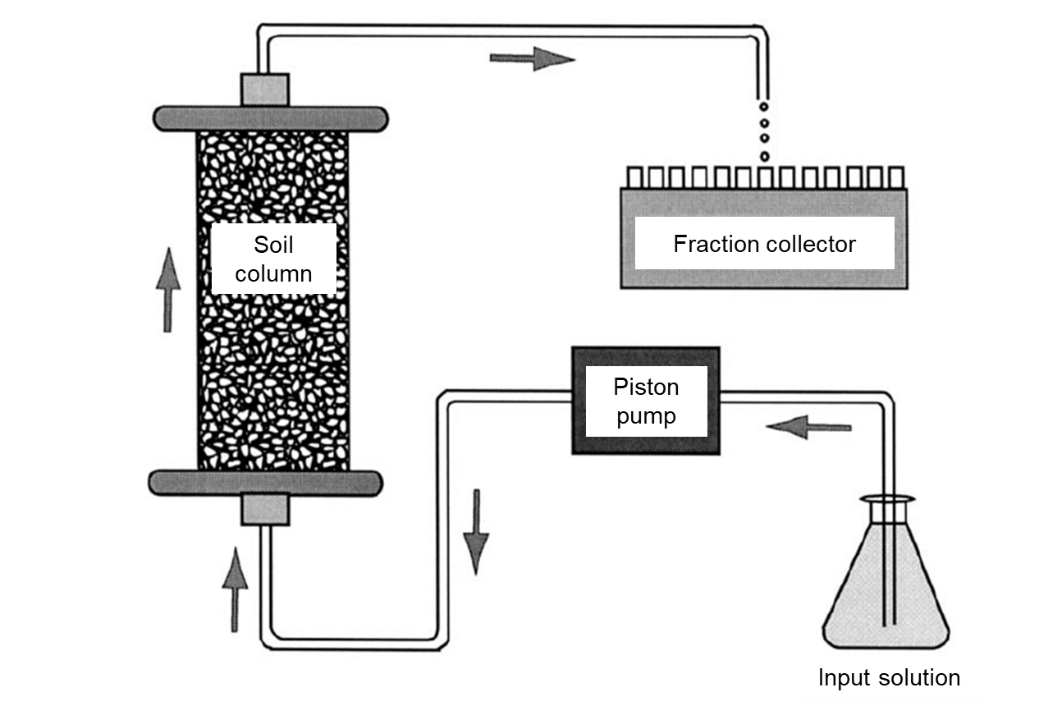
**

**Figure S1**. Schematic setup of saturated column system.

**Figure S2**. SEM images of (A1) original iron fillings, (A2) post-experiment iron fillings, (B1) original calcinated magnesite, (B2) post-experiment calcinated magnesite, (C1) original limestone, (C2) post-experiment limestone, (D1) original biochar, (D2) post-experiment biochar, collected from the inlet end of the packed columns.


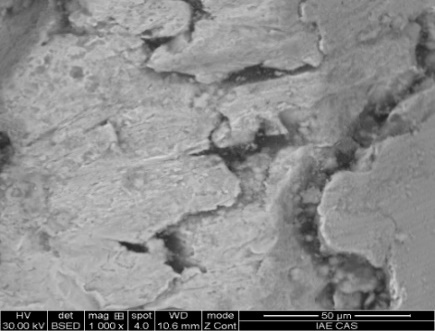

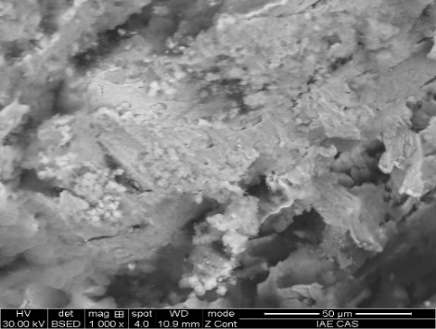

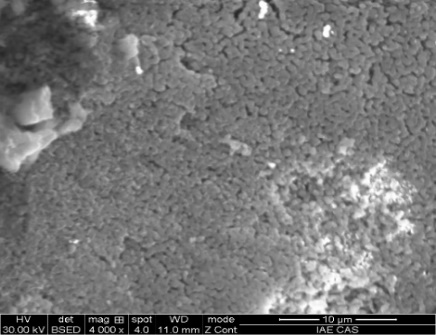

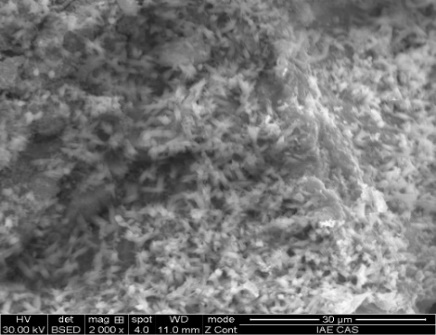

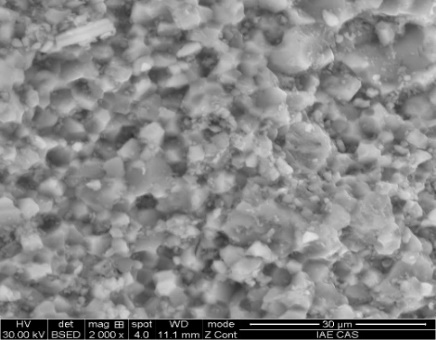

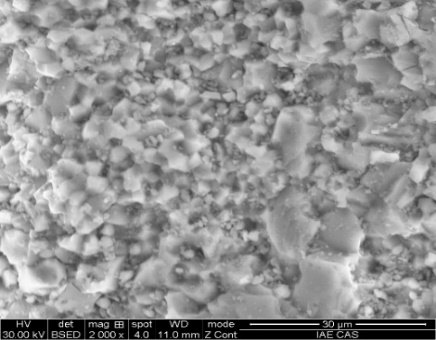

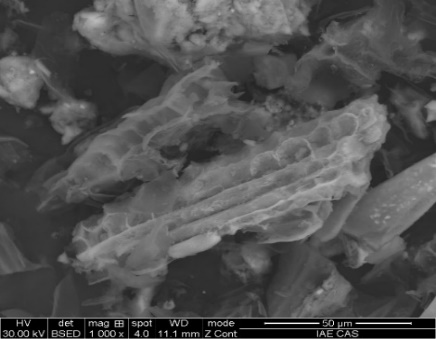

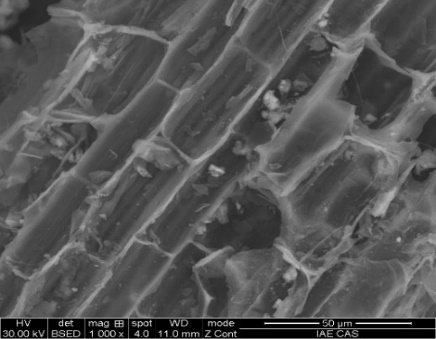


**(A1) Original iron filling**

**(A2) Post iron filling**

**50µm**

**×1000**

**50µm**

**×1000**

**50µm**

**×1000**

**50µm**

**×1000**

**50µm**

**×1000**

**50µm**

**×1000**

**50µm**

**×1000**

**50µm**

**×1000**

**(B1) Original calcinated**

**magnesite**

**(B2) Post calcinated magnesite**

**(C1) Original limestone**

**(C2) Post limestone**

**(D1) Original biochar**

**(D2) Post biochar**
